# Wearable Movement‐Tracking for Prodromal Parkinson's Disease Detection: A Cross‐Country Validation Study

**DOI:** 10.1002/mds.70329

**Published:** 2026-05-15

**Authors:** Fabian Kahl, Ann‐Kathrin Schalkamp, Katarina Mary Gunter, Anja Ophey, Sinah Röttgen, Jamil Razzaque, Konstantin Kufer, Cynthia Sandor, Gereon R. Fink, Elke Kalbe, Walter Maetzler, Clint Hansen, Stephan M. Jonas, Michele T. Hu, Lara Marie Reimer, Michael Sommerauer

**Affiliations:** ^1^ Institute for Digital Medicine, University of Bonn, University Hospital Bonn Bonn Germany; ^2^ Bakar Computational Health Sciences Institute University of California, San Francisco San Francisco California USA; ^3^ UK Dementia Research Institute at Imperial College London London UK; ^4^ Department of Brain Sciences Faculty of Medicine, Imperial College London London UK; ^5^ Nuffield Department of Clinical Neurosciences, Oxford Parkinson's Disease Centre University of Oxford Oxford UK; ^6^ Medical Psychology | Neuropsychology and Gender Studies, Center for Neuropsychological Diagnostics and Intervention (CeNDI), Faculty of Medicine and University Hospital Cologne, University of Cologne Cologne Germany; ^7^ Cognitive Neuroscience, Institute for Neuroscience and Medicine (INM‐3), Research Centre Juelich Juelich Germany; ^8^ Department of Neurology Faculty of Medicine and University Hospital Cologne, University of Cologne Cologne Germany; ^9^ Clinic for Parkinson's Disease, Sleep Disorders, and Movement Disorders University of Bonn, University Hospital Bonn Bonn Germany; ^10^ German Centre for Neurodegenerative Diseases (DZNE) Bonn Germany; ^11^ Department of Neurology University Medical Center Schleswig‐Holstein Campus Kiel and Kiel University Kiel Germany

## Abstract

**Background:**

Models trained on accelerometer data have been proposed for detecting prodromal Parkinson's disease (PD). However, uncertainties in diagnosis timing in the UK Biobank (UKBB) may affect generalizability to other cohorts.

**Objectives:**

The aim of the study was to evaluate the performance of previously published models for prodromal PD detection in other international cohorts.

**Methods:**

We applied the models to data from German and British cohorts of individuals with isolated or idiopathic rapid eye movement sleep behavior disorder and healthy controls. We compared hourly acceleration patterns and classification performance across cohorts.

**Results:**

The British cohort exhibited visually similar activity patterns to UK Biobank but weaker statistical differences and reduced model performance. The German cohort showed no significant group differences and lower performance. No pair of cohorts demonstrated statistical equivalence.

**Conclusions:**

Models trained on UK Biobank data may capture early clinical disease rather than universal prodromal markers. Prospective validation in well‐characterized cohorts is essential before clinical translation. © 2026 The Author(s). *Movement Disorders* published by Wiley Periodicals LLC on behalf of International Parkinson and Movement Disorder Society.

Early detection of Parkinson's disease (PD) remains a major clinical challenge, as diagnosis typically occurs after substantial neurodegeneration and functional impairment.^1^ Identifying individuals in the prodromal phase could enable earlier intervention and improve long‐term outcomes. Recently, wearable accelerometers have emerged as promising tools to identify subtle motor changes years before clinical diagnosis. Schalkamp et al demonstrated that machine learning models trained on wrist‐worn accelerometry data from the UK Biobank (UKBB) could distinguish individuals later diagnosed with PD from age‐ and sex‐matched healthy controls (HCs) up to 7 years before diagnosis. They defined prodromal PD as individuals who received a PD diagnosis two or more years after accelerometer data collection.^2^

In that study, prodromal PD was defined retrospectively based on electronic health record (EHR) diagnosis dates. However, recent work indicates that PD diagnosis dates in UKBB are frequently delayed relative to clinical onset, often by several years.^3^ As a result, individuals labeled as prodromal may already exhibit early motor symptoms at the time of accelerometer assessment. The reported model performance may reflect the detection of early clinical disease rather than true prodromal states. This raises concerns regarding the generalizability of such models to prospectively characterized at‐risk cohorts. To address this, we validated the prodromal models using two independent cohorts: a German cohort^4^ and a British cohort^5^ composed of participants with isolated or idiopathic rapid eye movement sleep behavior disorder (iRBD) and HCs. Individuals with iRBD represent a well‐established prodromal population, with nearly 90% progressing to PD or dementia with Lewy bodies, characterized by equivalent parkinsonian motor features,^6^ and they already show early signs of motor deficits, particularly in quantitative motor assessments such as tapping, gait, and balance.^7^ These cohorts provide an opportunity to evaluate prodromal models using prospectively measured clinical disease stages.

## Methods

### Study Cohorts

The UKBB is a population‐based cohort of over 500,000 participants recruited between 2006 and 2010.^8^ Accelerometry data were collected between 2013 and 2015 using Axivity AX3 devices.^9^ PD diagnoses were obtained through linkage with National Health Service (NHS) EHRs, and Schalkamp et al defined an individual as having prodromal PD if their PD diagnosis occurred at least 2 years after the collection of accelerometry data.^2^ We used their five age‐ and sex‐matched prodromal Lasso logistic regression models for our experiments.

The German cohort was recruited through polysomnography‐confirmed iRBD screening in accordance with ICSD‐III criteria.^10^, ^11^ Participants were further characterized within the CogTrAiL‐RBD study.^4^, ^12^

The British cohort was recruited from the Oxford Parkinson's Disease Centre Discovery study.^5^ We only included participants with wrist‐worn accelerometry data.

Both the German and British cohorts wore Axivity AX6 devices on their dominant wrist and included individuals with iRBD, whom we consider prodromal PD.^5^, ^10^, ^12^

We obtained information on education and ethnic background for the included participants from the respective cohort coordinators on request. Education information was available for all cohorts, although it was recorded using different classification systems. Ethnic background information was available for the UKBB and British cohorts. Cohort descriptions are provided in Appendix A.

### Preprocessing

To be able to evaluate the German and British cohorts with the UKBB‐based prodromal models, we processed their raw acceleration data using the Oxford Wearables Group accelerometer package.^9^, ^13^, ^14^, ^15^ The framework standardized the signals and segmented them into 30‐second epochs. It derived activity categories (imputed, Moderate to Vigorous Physical Activity (MVPA), sedentary, sleep, and light) and summary statistics. It could not compute wear‐time bias–corrected features, resulting in five missing variables out of 81 (accounting for 6.7% ± 5.3% of the coefficients). We computed additional self‐derived features as described by Schalkamp et al.^2^

### Acceleration Analysis

We computed the hourly average acceleration for each group and cohort across all valid days, along with 95% confidence intervals (CIs). We assessed differences using Welch's *t*‐tests.

### Model Application and Evaluation

We applied the five Lasso logistic regression models developed by Schalkamp et al,^2^ which they trained on age‐ and sex‐matched UKBB prodromal PD and HC data, to the German and British cohorts. We measured model performance using the area under the receiver operating characteristic curve (AUROC) with 95% CIs. We assessed performance differences using Welch's *t*‐tests for each pair of cohorts. We performed equivalence testing using two one‐sided tests (TOST).

### Feature‐Level Analysis

We selected the five most influential features from the original models, defined as those with the largest average absolute coefficient magnitudes, and assessed group differences between the German and British cohorts using Welch's *t*‐tests.

## Results

### Hourly Acceleration Patterns

**FIG. 1:**
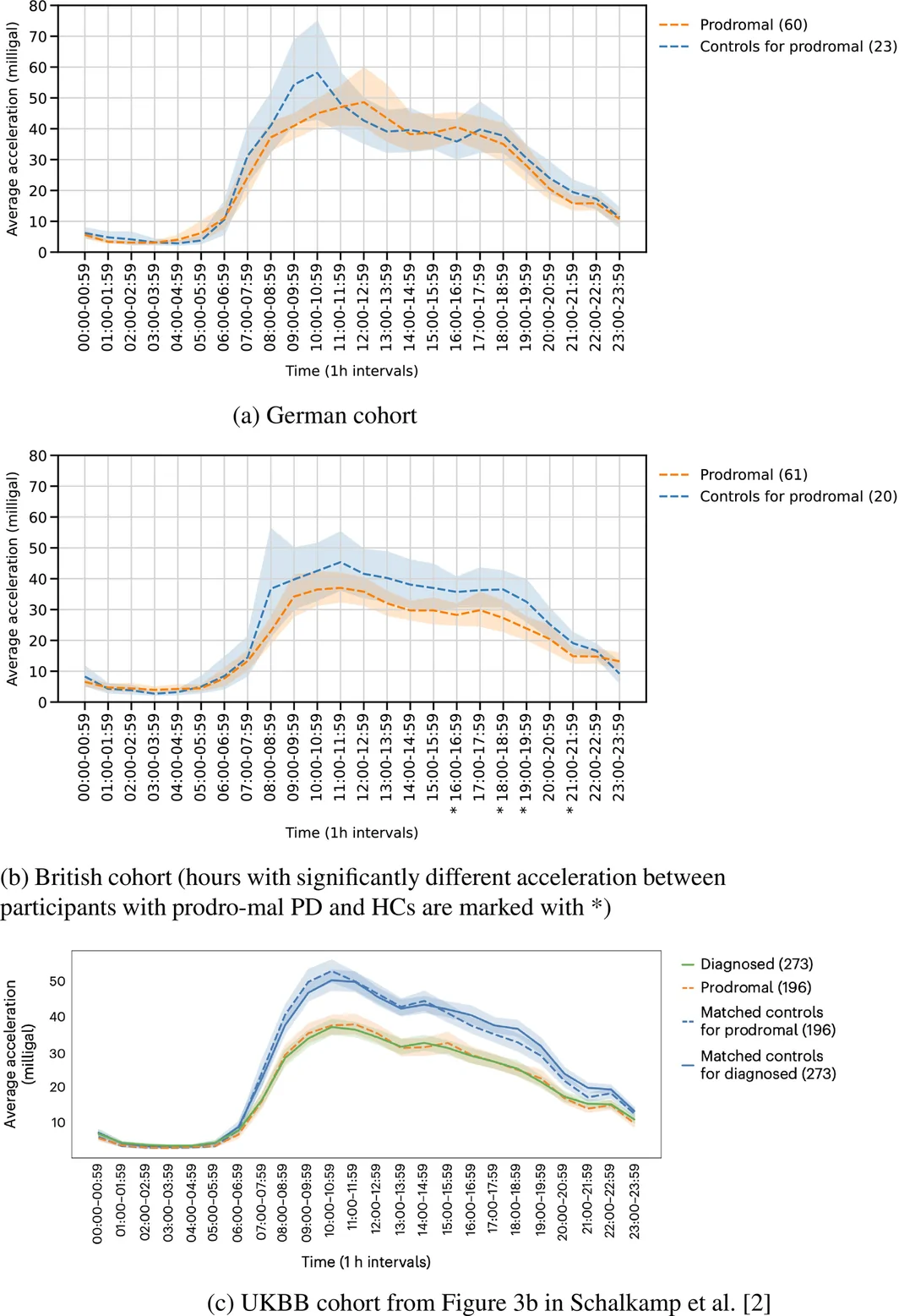
Average acceleration in 1‐hour intervals over all days with 95% confidence intervals. [Color figure can be viewed at wileyonlinelibrary.com]

In the UKBB cohort, participants with prodromal PD exhibited significantly lower average acceleration between 7 am and midnight compared to matched HCs (see Fig. 1C).^2^ In the German cohort, neither visual nor significant differences in hourly acceleration were observed between participants with prodromal PD and HCs (see Fig. 1A and Table A6). In the British cohort, we observed visually lower daytime acceleration in participants with prodromal PD than in HCs. However, statistically significant differences were limited to four nonconsecutive hours (see Fig. 1B and Table A7).

### Discrimination Performance

**FIG. 2:**
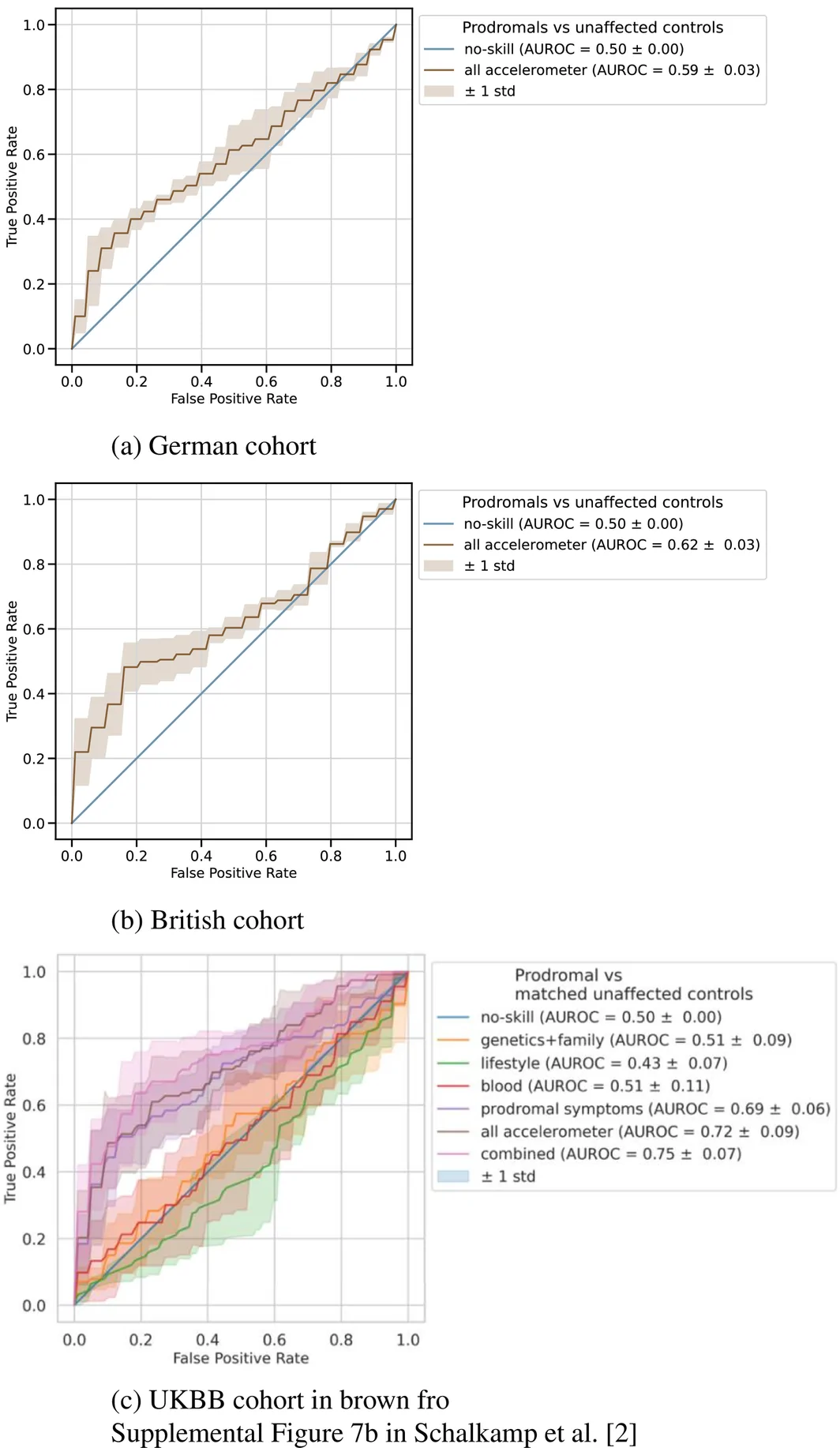
The mean receiver operator curves (ROC), along with their standard deviation, resulting from the five logistic regression models by Schalkamp et al,^2^ are shown for the three cohorts. [Color figure can be viewed at wileyonlinelibrary.com]

The original models achieved a reported AUROC of 0.72 ± 0.09 on the UKBB cohort (95% CI: 0.641–0.799).^2^ When applied to the German cohort, AUROC was 0.59 ± 0.03 (95% CI: 0.56–0.62; see Fig. 2A). In the British cohort, AUROC was 0.62 ± 0.03 (95% CI: 0.59–0.65; see Fig. 2B). Statistical comparisons using Welch's *t*‐test revealed significant performance differences between the UKBB and German cohorts (t‐statistic: 3.064, degrees of freedom (df): 4.878, *P*‐value: 0.029). In contrast, the performance difference between the UKBB and British cohorts, as well as between German and British cohorts, did not reach statistical significance using Welch's *t*‐test (t‐statistic: 2.357, df: 4.878, *P*‐value: 0.066, and t‐statistic: −1.581, df: 8.000, *P*‐value: 0.153, respectively).

Equivalence testing using TOST failed to demonstrate equivalence for any cohort pair (*δ* = 0.5,·SD = 0.03 [SD: overall standard deviation] and *α* = 0.05). For no pair of the three cohorts, equivalence was established (UKBB vs. German cohort: difference = 0.130, standard error [SE] = 0.042, df = 4.878, *P* lower = 0.007, *P*_upper = 0.967; UKBB vs. British cohort: difference = 0.100, SE = 0.042, df = 4.878, *P*_lower = 0.014, *P* upper = 0.919; and German vs. British cohort: difference = −0.030, SE = 0.022, df = 7.418, *P*_lower = 0.500, *P*_upper = 0.015).

### Feature‐Level Differences

The five most relevant features, ranked by average absolute coefficient, were the mean of the maximum consecutive sedentary hours per 24‐hour period, mean acceleration between 05:00 and 05:59 am, mean acceleration between 11:00 and 11:59 am, number of imputed intervals between 7 am and 11 pm, and number of light activity intervals between 11 pm and 7 am. None of them differed significantly between the groups in the German cohort (Welch's *t*‐test). In the British cohort, the most important feature was significantly higher in individuals with prodromal PD than in HCs. The other four features were not significantly different (*P* = 0.025; see Tables A8 and A9).

## Discussion

### Cultural Factors

The British cohort exhibited visually similar acceleration patterns to the UKBB cohort but showed weaker statistical differences between individuals with prodromal PD and HCs and reduced predictive performance. The German cohort showed neither significant group differences nor meaningful model performance. Cultural and behavioral differences in physical activity, transportation, and daily routines^16^ may influence accelerometry measures. However, cultural factors alone are insufficient to explain the observed discrepancies and likely interact with cohort design, recruitment strategies, and disease labeling practices.

### Diagnosis Timing

Recent evidence indicates that PD diagnosis dates in UKBB are frequently recorded several years after clinical diagnosis.^3^ Consequently, individuals classified as prodromal based on EHR linkage, as Schalkamp et al did,^2^ may already exhibit early motor symptoms at the time of accelerometer assessment, introducing label noise and inflating apparent effect sizes.

In contrast, participants in the German and British cohorts were prospectively recruited and systematically characterized. Individuals with prodromal PD were identified using polysomnography and longitudinal follow‐up, which provided more precise disease staging and reduced contamination from undiagnosed clinical PD.

Consistent with this distinction, the strong signal observed in UKBB, partial replication in the British cohort, and near absence of effects in the German cohort suggest that the patterns reported by Schalkamp et al^2^ may reflect early clinical disease rather than universal prodromal markers.

### Clinical Impact

Models trained on cohorts with uncertain timing of diagnosis may not generalize to clinically characterized at‐risk populations. Without prospective validation and precise disease staging, such models risk detecting early functional impairment rather than true prodromal disease. Population‐specific calibration and validation are therefore essential before clinical deployment.

### Limitations and Future Work

This study has several limitations. Sample sizes in the German and British cohorts were modest, limiting statistical power. Information on demographic diversity was limited. The UKBB and British cohorts were mainly composed of participants identifying as White, and ethnic background data were unavailable for the German cohort. Education levels varied across cohorts but were recorded using different classification systems, limiting direct comparability.

Future studies should include larger and more diverse populations and examine more complex activities, such as walking, to reduce potential cultural bias. We could not calculate wear‐time bias–corrected features, resulting in five missing features accounting for 6.7% ± 5.3% of model coefficients. Furthermore, because iRBD does not inevitably progress to PD, classification of these individuals as prodromal PD remains imperfect.

## Conclusion

We evaluated the generalizability of UKBB‐derived accelerometry‐based models for prodromal PD detection in two independent, prospective cohorts. Although the British cohort showed visually similar activity patterns to those in UKBB, neither cohort showed equivalent statistical differences or model performance. The German cohort showed no discernible prodromal signal. These findings suggest that delayed diagnosis recording in UKBB contributes to disease‐stage misclassification and inflated performance estimates, limiting transferability. Future studies should prioritize prospective cohorts with precise disease staging and account for lifestyle and demographic variability to improve robustness.

## Author Roles

1. Research Project: A. Conceptualization, B. Methodology, C. Software, D. Validation, E. Formal Analysis, F. Resources; 2. Manuscript Preparation: A. Writing – Original Draft, B. Writing – Review and Editing, C. Supervision, D. Funding Acquisition.

F.K.: 1A, 1B, 1C, 1D, 1E, 2A, 2B.

A.K.S.: 1D, 1F, 2B.

K.M.G.: 1C, 1F, 2B.

A.O.: 1F, 2B.

S.R.: 1F, 2B.

J.R.: 1F, 2B.

K.K.: 1F, 2B

C.S.: 1D, 1F, 2B

G.R.F.: 1F, 2B

E.K.: 1F, 2B

W.M.: 1F, 2B

C.H.: 1F, 2B

S.M.J.: 2B, 2C, 2D

L.M.R.: 1A, 1B, 1D, 2B, 2C, 2D

M.S.: 1A, 1B, 1F, 2B, 2C, 2D

## Financial Disclosures of All Authors (for the Past 12 Months)

The authors declare that there are no financial disclosures or conflicts of interest related to this work.

## Data Availability

The data that support the findings of this study are available on request from the corresponding author. The data are not publicly available due to privacy or ethical restrictions.

## Appendix A

### A.1 UK Biobank Cohort

The UK Biobank (UKBB) is a large‐scale biomedical database. It recruited 500,000 participants aged 40–69 years between 2006 and 2010 from across the United Kingdom. Participants were drawn from the National Health Service (NHS) patient registers, and 9 million individuals received invitations by mail. A total of 22 assessment centers were established across the United Kingdom to facilitate data collection.^8^ At the assessment centers, participants provided health and lifestyle information via questionnaires, underwent physical measurements (eg, height, weight, blood pressure, grip strength), and donated biological samples (eg, blood and urine). Their health records were subsequently linked to NHS electronic health records (EHRs) for long‐term follow‐up, tracking hospital admissions, disease diagnoses, and mortality outcomes.^17^ Between June 2013 and December 2015, the UKBB invited 236,519 participants to participate in an accelerometry study to measure physical activity objectively. The UKBB contacted participants who had provided a valid email address during the initial assessment for this phase. Of those invited, 106,053 agreed to wear a wrist‐worn triaxial accelerometer (Axivity AX3) on their dominant wrist continuously for 7 days, and 103,712 datasets were received. This device recorded raw acceleration data at 100 Hz, capturing movement information under free‐living conditions. The collected data were processed to derive physical activity metrics.^9^

Schalkamp et al defined an individual as prodromal if their Parkinson's disease (PD) diagnosis occurred at least 2 years after the collection of accelerometry data. For statistical analysis, they used data from 196 participants with prodromal PD (mean age: 69.25 ± 4.97 years, male: 69.39%) and 196 matched healthy controls (HCs) (mean age: 69.24 ± 4.96 years, male: 69.39%). To train the five Lasso logistic regression models, they included 113 participants with prodromal PD (mean age: 69.28 ± 4.72 years, male: 80.53%), and age‐ and sex‐matched HCs.^2^

Information on education and ethnic background was provided by the cohort coordinators upon request. They coded education into degree, postsecondary education, secondary education, no formal qualifications, and missing information. Among HCs, 13 participants reported no formal qualifications, 13 reported secondary education, 32 reported postsecondary education, and 54 reported a degree, with education information missing for 1 participant. Among individuals with prodromal PD, 14 participants reported no formal qualifications, 11 reported secondary education, 40 reported postsecondary education, and 47 reported a degree, with education information missing for 1 participant (see Table A1).

They provided ethnic background information according to data‐field 21000 of the UKBB. The majority of participants in both groups identified as White, predominantly White British. In the HC group, 99 participants identified as White British, with smaller numbers identifying as Irish (3), White with no further specification (1), other White backgrounds (4), Asian or Asian British (2), Chinese (1), mixed background (1), or preferring not to answer (2). Among individuals with prodromal PD, 104 participants identified as White British, with smaller numbers identifying as Irish (4), other White backgrounds (2), Asian or Asian British (2), or preferring not to answer (1; see Table A2).

### A.2 German Cohort

Between June 2020 and July 2021, Seger et al conducted a public recruitment campaign via graphical advertisements in local newspapers around the University Hospital Cologne, Germany. They invited 124 individuals for video‐polysomnography to confirm an isolated or idiopathic rapid eye movement sleep behavior disorder (iRBD) diagnosis according to the International Classification of Sleep Disorders III criteria.^10^, ^11^

Ophey et al recruited participants from this continuously expanding cohort into the ongoing CogTrAiL‐RBD study. They provided them with an Axivity AX6 accelerometer, recording data at 100 Hz on their dominant wrist for seven consecutive days. Additional inclusion criteria for individuals with iRBD included age between 40 and 80 years, normal or corrected‐to‐normal vision and hearing, and proficiency in German, with exclusions for significant cognitive impairment (Montreal Cognitive Assessment ≤ 22) or major neurological/psychiatric disorders. They recruited HCs via newsletters and flyers, using the same inclusion and exclusion criteria and excluding diagnoses of movement disorders, signs of iRBD, or any other psychiatric or neurological condition.^4^, ^12^ For the present data analysis, we included data collected between June 2022 and April 2024. We additionally excluded HCs with signs of mild cognitive impairment, as assessed by Level II criteria according to the Movement Disorder Society,^18^ in the neuropsychological baseline assessment of CogTrAiL‐RBD, resulting in the exclusion of four originally recruited HCs.

In addition, we received data from three additional HCs and eight individuals with iRBD from Michael Sommerauer's consultation hour, who met the same criteria as the CogTrAiL‐RBD participants. One individual with iRBD was already included in the CogTrAiL‐RBD dataset. This resulted in a final cohort of 23 HCs (mean age: 67.74 ± 4.97 years, 91.30% male) and 60 participants with iRBD (mean age: 68.73 ± 5.66 years, 83.33% male).

Information on education was provided by the cohort coordinators upon request. Education was coded using categories that reflect the German education and vocational training system. These included vocational training (Berufliche Ausbildung), vocational training and university of applied sciences degree (Berufliche Ausbildung und Fachhochschulstudium), university of applied sciences degree (Fachhochschulstudium), university degree (Hochschulstudium), university degree and postgraduate qualifications (Hochschulstudium und Postgraduale Qualifikation), and missing. Among HCs, the most frequent categories were vocational training (eight participants) and university degrees (eight participants), with smaller numbers in other categories. Among participants with iRBD, vocational training (24 participants) and university degrees (16 participants) were the most common categories, with additional representation across the other educational categories (see Table A3). Information on ethnic background was not available for the German cohort.

### A.3 British Cohort

The Oxford Parkinson's Disease Centre (OPDC) Discovery cohort is a prospective, longitudinal study on the progression of PD. Initiated in September 2010, it recruits individuals with early idiopathic PD, HCs, at‐risk individuals (first‐degree relatives), and individuals with polysomnography‐confirmed iRBD. At baseline, the cohort included 1082 individuals with PD, 297 HCs, 106 PD relatives, and 104 individuals with iRBD. Inclusion criteria for individuals with iRBD were as follows: informed consent, fluency in English, being at least 18 years old, and no evidence of significant cognitive impairment. They selected HCs to match the age and sex distribution, often drawn from spouses or friends. Exclusion criteria for individuals with iRBD included significant cognitive impairment, nonidiopathic or secondary parkinsonism, atypical parkinsonian syndromes, or dementia preceding PD by 1 year. HCs with a family history of PD, stroke, substance abuse, significant disability, or extrapyramidal signs were excluded.^5^

All participants in the OPDC Discovery cohort were invited to take part in substudies, such as the Axivity study. Initially, the participants wore the Axivity device on their lumbar. They later changed to wrist placement. Only wrist data are included in this study, leading to 61 participants with iRBD (mean age: 69.01 ± 8.08 years, 70.49% male) and 20 HCs (mean age: 70.90 ± 9.00 years, 40.00% male).

Information on education and ethnic background was provided by the cohort coordinators upon request. They categorized education into 12 years or less, more than 12 years, and missing. Among HCs, 5 participants reported ≤ 12 years, 13 reported > 12 years, and 2 had missing data. Among individuals with iRBD, 17 reported ≤ 12 years and 44 reported > 12 years (see Table A4).

Most participants identified as White. Among HCs, 18 participants were recorded as White, and 2 had missing ethnic information. Among individuals with iRBD, 60 participants were recorded as White, and 1 participant was recorded as non‐White (see Table A5).

**TABLE A1:** Education distribution in the UK Biobank (UKBB) cohort used in this study

Status	None	Secondary	Postsecondary	Degree	Missing
HCs	13	13	32	54	1
Prodromal PD	14	11	40	47	1

Abbreviations: HCs, healthy controls; PD, Parkinson's disease.

**TABLE A2:** Ethnic background distribution in the UK Biobank (UKBB) cohort used in this study

Status	Ethnic background (general)	Ethnic background (specific)	Count
HCs	White	British	99
HCs	White	Irish	3
HCs	White	Not specified	1
HCs	White	Any other white background	4
HCs	Asian or Asian British	Pakistani	1
HCs	Asian or Asian British	Any other Asian background	1
HCs	Chinese	Chinese	1
HCs	Mixed	Any other mixed background	1
HCs	Prefer not to answer	Prefer not to answer	2
iRBD	White	British	104
iRBD	White	Irish	4
iRBD	White	Any other white background	2
iRBD	Asian or Asian British	Indian	2
iRBD	Prefer not to answer	Prefer not to answer	1

Abbreviations: HCs, healthy controls; iRBD, isolated or idiopathic rapid eye movement sleep behavior disorder.

**TABLE A3:** Education distribution in the German cohort

Status	Education	Count
HCs	Vocational training (Berufliche Ausbildung)	8
HCs	Vocational training and university of applied sciences degree (Berufliche Ausbildung und Fachhochschulstudium)	1
HCs	University of applied sciences degree (Fachhochschulstudium)	1
HCs	University degree (Hochschulstudium)	8
HCs	University degree and postgraduate qualification (Hochschulstudium und postgraduale Qualifikation)	2
HCs	Missing	3
iRBD	None (Keine)	2
iRBD	Vocational training (Berufliche Ausbildung)	24
iRBD	Vocational training and university of applied sciences degree (Berufliche Ausbildung und Fachhochschulstudium)	2
iRBD	University of applied sciences degree (Fachhochschulstudium)	3
iRBD	University degree (Hochschulstudium)	16
iRBD	University degree and postgraduate qualification (Hochschulstudium und postgraduale Qualifikation)	5
iRBD	Missing	8

Note: German education categories are translated with the original term in parentheses. Abbreviations: HCs, healthy controls; iRBD, isolated or idiopathic rapid eye movement sleep behavior disorder.

**TABLE A4:** Education distribution in the British cohort

Status	≤ 12 y	> 12 y	Missing
HCs	5	13	2
iRBD	17	44	0

Abbreviations: HCs, healthy controls; iRBD, isolated or idiopathic rapid eye movement sleep behavior disorder.

**TABLE A5:** Ethnic background distribution in the British cohort

Status	White	Non‐white	Missing
HCs	18	0	2
iRBD	60	1	0

Abbreviations: HCs, healthy controls; iRBD, isolated or idiopathic rapid eye movement sleep behavior disorder.

**TABLE A6:** Welch's t‐test between 60 participants with prodromal Parkinson's disease (PD) and 23 healthy controls (HCs) across hourly intervals in the German cohort

Hour	T	df	Alt.	*P*‐value	95% CI	Cohen's *d*	BF_10_	Power
∅ 00:00–00:59	0.573	44	Two‐sided	0.570	[−1.45, 2.61]	0.133	0.289	0.083
∅ 01:00–01:59	1.743	25	Two‐sided	0.093	[−0.26, 3.17]	0.584	0.906	0.653
∅ 02:00–02:59	1.002	23	Two‐sided	0.327	[−1.12, 3.23]	0.369	0.385	0.319
∅ 03:00–03:59	0.208	33	Two‐sided	0.837	[−0.99, 1.22]	0.056	0.256	0.056
∅ 04:00–04:59	−1.594	77	Two‐sided	0.115	[−2.64, 0.29]	0.262	0.736	0.185
∅ 05:00–05:59	−1.328	74	Two‐sided	0.188	[−6.02, 1.20]	0.214	0.531	0.139
∅ 06:00–06:59	−0.121	38	Two‐sided	0.905	[−7.28, 6.46]	0.030	0.253	0.052
∅ 07:00–07:59	1.245	45	Two‐sided	0.220	[−4.31, 18.24]	0.288	0.485	0.213
∅ 08:00–08:59	0.690	32	Two‐sided	0.495	[−7.41, 15.02]	0.189	0.308	0.118
∅ 09:00–09:59	1.864	26	Two‐sided	0.073	[−1.36, 28.05]	0.605	1.088	0.684
∅ 10:00–10:59	1.470	25	Two‐sided	0.154	[−5.23, 31.39]	0.490	0.627	0.506
∅ 11:00–11:59	0.193	40	Two‐sided	0.848	[−11.13, 13.48]	0.047	0.255	0.054
∅ 12:00–12:59	−0.949	78	Two‐sided	0.346	[−18.42, 6.53]	0.172	0.368	0.107
∅ 13:00–13:59	−0.719	77	Two‐sided	0.474	[−16.21, 7.61]	0.131	0.313	0.083
∅ 14:00–14:59	0.289	52	Two‐sided	0.774	[−8.18, 10.93]	0.063	0.260	0.057
∅ 15:00–15:59	−0.127	66	Two‐sided	0.899	[−8.24, 7.25]	0.025	0.253	0.051
∅ 16:00–16:59	−1.101	46	Two‐sided	0.276	[−13.25, 3.88]	0.250	0.420	0.172
∅ 17:00–17:59	0.372	38	Two‐sided	0.712	[−8.56, 12.42]	0.093	0.267	0.066
∅ 18:00–18:59	0.617	73	Two‐sided	0.539	[−6.06, 11.49]	0.116	0.295	0.076
∅ 19:00–19:59	0.661	77	Two‐sided	0.511	[−4.80, 9.57]	0.121	0.303	0.077
∅ 20:00–20:59	1.138	42	Two‐sided	0.261	[−2.74, 9.84]	0.269	0.435	0.192
∅ 21:00–21:59	1.692	38	Two‐sided	0.099	[−0.72, 8.14]	0.421	0.842	0.396
∅ 22:00–22:59	0.660	42	Two‐sided	0.513	[−2.90, 5.72]	0.157	0.302	0.097
∅ 23:00–23:59	0.247	35	Two‐sided	0.806	[−3.50, 4.47]	0.065	0.258	0.058

Abbreviations: df, degrees of freedom; Alt., alternative; CI, confidence interval.

**TABLE A7:** Welch's t‐test between 61 participants with prodromal Parkinson's disease (PD) and 20 healthy controls (HCs) across hourly intervals in the British cohort

Hour	T	df	Alt.	*P*‐value	95% CI	Cohen's *d*	BF_10_	Power
∅ 00:00–00:59	0.952	26	Two‐sided	0.350	[−2.02, 5.52]	0.283	0.383	0.192
∅ 01:00–01:59	−0.416	37	Two‐sided	0.680	[−2.29, 1.51]	0.099	0.282	0.067
∅ 02:00–02:59	−0.641	50	Two‐sided	0.525	[−2.60, 1.34]	0.133	0.311	0.080
∅ 03:00–03:59	−2.496	78	Two‐sided	0.015	[−2.20, −0.25]	0.412	3.439	0.353
∅ 04:00–04:59	−1.390	73	Two‐sided	0.169	[−2.51, 0.45]	0.248	0.587	0.158
∅ 05:00–05:59	0.314	22	Two‐sided	0.756	[−2.80, 3.81]	0.108	0.273	0.070
∅ 06:00–06:59	0.249	31	Two‐sided	0.805	[−5.55, 7.09]	0.065	0.269	0.057
∅ 07:00–07:59	0.323	29	Two‐sided	0.749	[−6.28, 8.64]	0.089	0.274	0.063
∅ 08:00–08:59	1.497	21	Two‐sided	0.149	[−5.33, 32.76]	0.566	0.668	0.583
∅ 09:00–09:59	0.857	39	Two‐sided	0.397	[−7.56, 18.68]	0.199	0.356	0.119
∅ 10:00–10:59	1.071	34	Two‐sided	0.292	[−5.42, 17.52]	0.268	0.424	0.177
∅ 11:00–11:59	1.538	30	Two‐sided	0.134	[−2.72, 19.42]	0.408	0.704	0.346
∅ 12:00–12:59	1.251	31	Two‐sided	0.220	[−3.64, 15.21]	0.329	0.504	0.243
∅ 13:00–13:59	1.766	28	Two‐sided	0.088	[−1.31, 17.74]	0.497	0.961	0.479
∅ 14:00–14:59	1.973	25	Two‐sided	0.059	[−0.35, 17.09]	0.598	1.324	0.631
∅ 15:00–15:59	1.534	30	Two‐sided	0.135	[−2.40, 16.90]	0.414	0.700	0.355
∅ 16:00–16:59	2.421	39	Two‐sided	0.020	[1.23, 13.68]	0.560	2.963	0.574
∅ 17:00–17:59	1.482	51	Two‐sided	0.145	[−2.30, 15.26]	0.307	0.656	0.218
∅ 18:00–18:59	2.284	46	Two‐sided	0.027	[1.10, 17.41]	0.493	2.278	0.472
∅ 19:00–19:59	2.114	29	Two‐sided	0.043	[0.28, 17.04]	0.583	1.676	0.608
∅ 20:00–20:59	1.446	49	Two‐sided	0.154	[−1.87, 11.46]	0.303	0.628	0.213
∅ 21:00–21:59	2.045	35	Two‐sided	0.048	[0.03, 8.42]	0.501	1.490	0.484
∅ 22:00–22:59	1.126	53	Two‐sided	0.265	[−1.47, 5.25]	0.229	0.446	0.142
∅ 23:00–23:59	−1.815	43	Two‐sided	0.076	[−8.41, 0.44]	0.402	1.033	0.338

Abbreviations: df, degrees of freedom; Alt., alternative; CI, confidence interval.

**TABLE A8:** Welch's t‐test between prodromal and healthy groups across top five features of the five logistic regression models in the German cohort

Feature	T	df	Alt.	*P*‐value	95% CI	Cohen's *d*	BF_10_	Power
∅ max. sedentary h. consec. per 24 h	−0.416	58	Two‐sided	0.679	[−0.42, 0.27]	0.086	0.271	0.064
∅ 05:00–05:59	−1.328	74	Two‐sided	0.188	[−6.02, 1.20]	0.214	0.531	0.139
∅ 11:00–11:59	0.193	40	Two‐sided	0.848	[−11.13, 13.48]	0.047	0.255	0.054
∅ No. imputed intervals 07–23	−1.182	45	Two‐sided	0.243	[−0.09, 0.02]	0.273	0.454	0.196
∅ No. light intervals 23–07	0.818	33	Two‐sided	0.419	[−0.30, 0.71]	0.220	0.334	0.144

Abbreviations: max., maximum; h., hours; consec., consecutive; No., number; df, degrees of freedom; Alt., alternative; CI, confidence interval.

**TABLE A9:** Welch's t‐test between prodromal and healthy groups across top five features of the five logistic regression models in the British cohort

Feature	T	df	Alt.	*P*‐value	95% CI	Cohen's *d*	BF_10_	Power
∅ max. sedentary h. consec. per 24 h	−2.326	39	Two‐sided	0.025	[−0.93, −0.06]	0.531	2.480	0.514
∅ 05:00–05:59	0.314	22	Two‐sided	0.756	[−2.80, 3.81]	0.108	0.273	0.070
∅ 11:00–11:59	1.538	30	Two‐sided	0.134	[−2.72, 19.42]	0.408	0.704	0.346
∅ No. imputed intervals 07–23	0.806	19	Two‐sided	0.430	[−0.49, 1.11]	0.336	0.349	0.244
∅ No. light intervals 23–07	1.655	25	Two‐sided	0.110	[−0.16, 1.44]	0.488	0.830	0.450

Abbreviations: max., maximum; h., hours; consec., consecutive; No., number; df, degrees of freedom; Alt., alternative; CI, confidence interval.
